# A Personalized Medical Decision Support System Based on Explainable Machine Learning Algorithms and ECC Features: Data from the Real World

**DOI:** 10.3390/diagnostics11091677

**Published:** 2021-09-14

**Authors:** Dongxiao Gu, Wang Zhao, Yi Xie, Xiaoyu Wang, Kaixiang Su, Oleg V. Zolotarev

**Affiliations:** 1 The School of Management, Hefei University of Technology, Hefei 230009, China; 2019110768@mail.hfut.edu.cn (W.Z.); yixie928@163.com (Y.X.); 2018110745@mail.hfut.edu.cn (K.S.); 2 Key Laboratory of Process Optimization and Intelligent Decision-Making of Ministry of Education, Hefei 230009, China; 3 The 1st Affiliated Hospital, Anhui University of Traditional Chinese Medicine, Hefei 230009, China; xywang0551@163.com; 4 The Department of Information Systems in Economics and Management, Russian New University, 105005 Moscow, Russia; ol-zolot@yandex.ru

**Keywords:** case-based reasoning, personalized recommendations, machine learning, external features of cases, physician adoption

## Abstract

Artificial intelligence can help physicians improve the accuracy of breast cancer diagnosis. However, the effectiveness of AI applications is limited by doctors’ adoption of the results recommended by the personalized medical decision support system. Our primary purpose is to study the impact of external case characteristics (ECC) on the effectiveness of the personalized medical decision support system for breast cancer assisted diagnosis (PMDSS-BCAD) in making accurate recommendations. Therefore, we designed a novel comprehensive framework for case-based reasoning (CBR) that takes the impact of external features of cases into account, made use of the naive Bayes and k-nearest neighbor (KNN) algorithms (CBR-ECC), and developed a PMDSS-BCAD system by using the CBR-ECC model and external features as system components. Under the new case-based reasoning framework, the accuracy of the combined model of naive Bayes and KNN with an optimal K value of 2 is 99.40%. Moreover, in a real hospital scenario, users rated the PMDSS-BCAD system, which takes into account the external characteristics of the case, better than the original personalized system. These results suggest that PMDSS-BCD can not only provide doctors with more personalized and accurate results for auxiliary diagnosis, but also improve doctors’ trust in the results, so as to encourage doctors to adopt the results recommended by the personalized system.

## 1. Introduction

The International Agency for Research on Cancer of the World Health Organization has released the latest global cancer data for 2020, and there were about 2.3 million new cases of breast cancer worldwide in 2020, accounting for nearly 12% of all cancer cases, surpassing lung cancer as the most prevalent cancer type worldwide for the first time [1]. With the improvement of living standards, the accelerated pace of life, and the increase in life pressures and stress, the incidence of breast diseases, especially breast cancer, is also increasing, and the age of affected individuals is getting younger [2]. It is predicted that by 2030, cancer incidence will affect more than half of the population. Moreover, while survival rates are 89% in the United States and 76% in Europe, in developing countries survival rates are dropping [3]. Thus, the management of breast cancer remains one of the most problematic healthcare issues [4].

In this context, machine learning represents a great opportunity for:

(1) Supporting physicians, biologists, and medical authorities to develop and significantly improve big medical data analytics;
(2) Reducing the risk of medical errors;
(3) Better coordinating diagnostic and prognostic options [5,6].

A revolution in medicine driven by artificial intelligence is taking place, with a large number of new methods being introduced to improve the accuracy of diagnosis in breast cancer management and to provide new solutions for breast cancer diagnosis [7]. Mangasarian et al. used methods such as machine learning to achieve highly accurate diagnosis and prognosis of breast cancer [8]. Liu et al. developed a support vector machine with better performance in breast cancer diagnosis [9]. Akay proposed a breast cancer diagnosis based on a combination of a support vector machine and a feature selection method, and was able to obtain higher classification accuracy than previous methods [10]. Vanitha et al. studied five AI methods for managing breast cancer, such as using a support vector machine, and evaluated their performances [11]. Alaa et al. used large-scale data from cohorts to develop a breast cancer prognostication and treatment benefit prediction model [12]. Therefore, new strategies for accurate and effective ongoing management using AI are highly desirable [13].

Decision support systems for disease management using machine learning have been used to some extent in the medical community [14,15]. However, the personalized-system-recommended results are often not recognized by the physician community, making it difficult to exploit the advantages of AI in improving the quality of healthcare and reducing the burden on medical resources [16,17,18], a problem that deserves reflection by AI practitioners. In this regard, medical experts are currently facing two challenging problems:

1. Previous studies focused on improving the diagnostic accuracy, and it was seldom considered whether the results with high diagnostic accuracy recommended by the personalized system could be trusted and adopted by doctors.
2. The diagnostic features considered in previous studies are all internal features of the case itself, and the prior knowledge from case experts, such as the external features of the case, is rarely considered; these external features are equally important for the personalized systematic results [19].

Therefore, based on the above research status, this study proposes a novel CBR framework and establishes a CBR model that considers the effects of external characteristics (CBR-ECC model) and the personalized medical decision support system for breast cancer assisted diagnosis (PMDSS-BCAD) system [20]. In this new CBR framework [21,22], naive Bayes and the KNN model, both used in the retrieval stage, aimed to verify the case retrieval algorithm to improve the performance of CBR system, and the integration of the ECC [23,24] is used to improve the trust and adoption of the recommended results by medical staff. This study provides health care professionals with a technique for personalized diagnosis of breast cancer that is not only reliable but also credible. It helps to provide supporters and future researchers with more literature on the impact of global breast cancer treatment [25].

The rest of this paper is organized as follows. In the next section, we present the data from the Mozambique dataset and describe the methods and processes for feature processing, naive Bayes classification, KNN retrieval, and ECC feature fusion of the data. In Section 3, we show the test results of each phase of the models and methods, and the evaluation of the application of the decision support system in a real hospital scenario. Finally, we summarize the critical discussion and conclusions of this paper.

## 2. Materials and Methods

### 2.1. Data Description and Model Implementation

**Figure 1:**
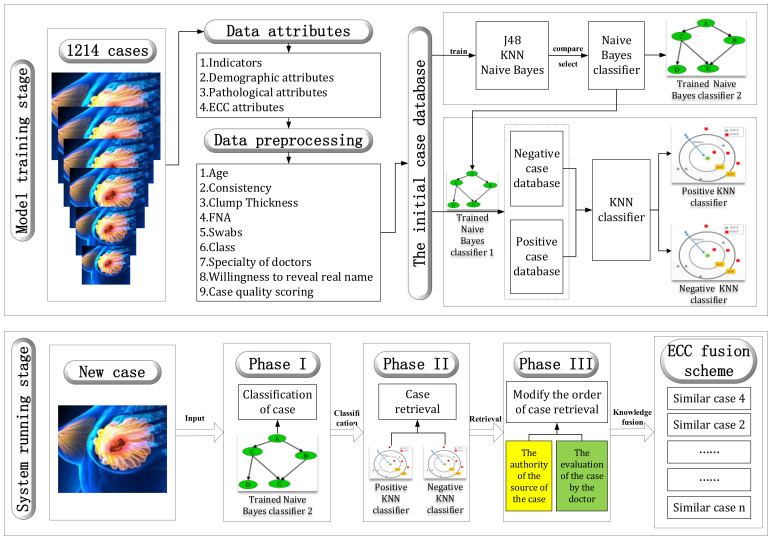
PMDSS-BCAD system framework.

The conceptual framework of the PMDSS-BCAD system consists of three main components as shown in Figure 1. The first stage is to use naive Bayes to evaluate whether the patients who begin use of the system have malignant or benign tumors, so as to realize the classification of positive and negative cases of patients. In the second stage, the k-nearest neighbor algorithm (KNN) with the best K value on the base of the corresponding category of new patients in the first stage is implemented to obtain the retrieval cases with high similarity. Although KNN was chosen as our improved search process algorithm, the application of an additional classifier before KNN can further improve the results. This explains why the first stage of the PMDSS-BCAD system framework is set up. The third stage is to fuse the diagnostic results achieved by naive Bayes and KNN in the second stage with the ECC and correct the ranking of similar medical records in order to make the final results more consistent with the clinical, logical way of thinking that doctors employ, and to promote the adoption of the results by physicians.

#### 2.1.1. Data Description

The following is the data description. We analyzed the provided data and preprocessed it to fit the experiment. In the study, we used an all-labeled dataset consisting of about 1200 cases from the database of the Maputo Central Hospital (HCM) in Mozambique, which initially contained 25 attributes (including indicators, demographic attributes, pathological attributes, and ECC attributes), was used for this study. We decided to test our algorithm on real datasets to ensure its validity. 

The methods used by pathologists to examine fine-needle aspirate (FNA) tissue specimens of breast cancer are mainly divided into macroscopic and microscopic analysis. They study the outer appearance of the lesion where the tumor is and pay close attention to the characteristics of the cells. Cancer, commonly known as a tumor, is usually caused by the uncontrolled division of cells into distinct masses. Tumors can be benign or malignant, and the latter is detrimental as it may develop rapidly and spread over the nearby tissues [26]. Our selected sample dataset initially contained 950 benign cases and 264 malignant cases. The attribute category has two values, “1” for malignant cancer and “0” for benign cancer. As the data are extensive and some information does not influence patients’ condition or diagnosis, preprocessing is needed.

Data preprocessing is an important step in data mining. It transforms the original data into a format more suitable for machine understanding. In the real world, there are always some problems with data. In order to get accurate, complete, and consistent data, data preprocessing is necessary. It is applied to make data more suitable for analysis [27,28]. In our study, we used the following approaches for data preprocessing: dimensionality reduction (feature subset selection), discretization and binarization, variable transformation, and feature creation.

##### Dimensionality Reduction/Feature Subset Selection

Feature subset selection or feature selection is a method of dimensionality reduction that selects attributes that belong to the subset of a former attribute and makes them new attributes. We used filter approaches for this feature subset selection and chose sets of attributes with low pairing correlation. Initially the following attributes were taken into account: age, gender, macro-analysis, micro-analysis, head (main location of the tumor), location 1, location 2, doctor’s education and title, doctor’s real name (the relevant literature has shown that these two doctor-related attributes reflect authoritative information of the physician, but as privacy is involved, the relevant information will be used after desensitization [29]), the remarks of the case (including case quality information), and the final diagnosis. The remaining attributes were discarded.

##### Discretization and Binarization

The process of transforming continuous attributes into classified attributes is discretization. The process that converts continuous and discrete attributes into a single or multiple binary attributes is called binarization. When a categorical attribute contains big-value numbers, or some values that appear infrequently, discretization and/or binarization can be beneficial to decrease the amount of categories through the combination of a few values. The attributes mentioned here are binarized. Not all attributes are binarized because such a conversion can cause hurdles, for instance, undesirable relationships would be generated in the converted attributes.

##### Variable Transformation

Standardization or normalization is a common type of variable transformation [28]. In order to achieve a normalized process, redundant data need to be eliminated and meaningful data dependencies ensured. We normalize all values in a given dataset (except for the class attribute, if set). In general, the results of normalized data are within the interval [0, 1]. In special cases, tools such as scale and shift parameters can be used for processing. For example, when scale = 2.0 and shift = −1.0, values in the range of (−1, +1) will be achieved.

##### Feature Creation

New features can be created from existing attributes. This new group of features (that is, new attributes) can replace the originals and effectively highlight the most important characteristics in a dataset, which has the advantage of dimensionality reduction by having fewer attributes. The approach used for feature creation in this study is feature extraction. The information contained in the macroscopic analysis, namely clump thickness, location, consistency, set (i.e., mobility of the clump), and the type of FNA material extracted, is all useful information. Since the combination of all these details would give us a complex dataset, we performed a feature extraction and analyzed each item one by one, contributing to a better and well-focused specific analysis. We also made swabs, stained them, and created a description of cell attributes for the microscopic analysis. In addition to pathology features, we created features related to ECC (e.g., specialty of doctors, willingness to reveal real name, case quality scoring) that influenced our final results. The final diagnosis is set as an attribute. Based on the final diagnosis, the attribute class was created, which divided the data into two distinct groups: patients with a malign tumor or a benign tumor. Of the 25 attributes in the dataset, the rest of the attributes, namely index, gender, race, bed, and dates of analysis, were discarded, as they did not apply in any way to the final diagnosis.

In this section, we present and explain in detail all the approaches used in the study. All of the steps mentioned helped reduce the number of attributes in our dataset, making our model more understandable and easier to visualize, and reduced the process time of the algorithm. Based on the dimensionality reduction approach, we combined some attributes and removed some to reduce the noise data in our set. The data are abundant, but not all attributes are needed to achieve the purpose of our study. We retained the features shown in Table 1; the attribute descriptions are shown in Table 2.

#### 2.1.2. Descriptive Model (Phase I)

To build the descriptive model and obtain cases for knowledge representation, three descriptive models involving naive Bayes, the KNN algorithm, and decision tree classifiers were constructed and evaluated. Naive Bayes, the adopted classifier, is the focus in this study. It deals with the following two main tasks: (1) First, to classify the database of cases using the naive Bayes model, that is, we classify all cases in the initial case database into malignant and benign case sub-databases, providing the case database of the corresponding category for KNN retrieval later on. (2) Second, to classify new patients entering the PMDSS-BCAD system using naive Bayes; that is, whenever a new breast cancer case is analyzed, an assessment of whether this new breast cancer case has a malignant or benign tumor is performed to achieve the classification of this patient. The Bayesian theorem frames the reason for ascertaining the probabilities of theories, the foundation of the Bayes classifier, and the premise of algorithms for assessing estimations of imperceptibility factors. The Bayesian classifier is found to be practically identical in execution with the decision tree and neural network classifiers. Bayesian classifiers in correlation with others have additionally shown high exactness and speed when connected to huge databases. These classifiers expect that the impact of a characteristic incentive on a given class is autonomous of the estimations of alternate qualities. This suspicion is called class-contingent independence. It is made to disentangle the calculations included and, in this sense, is viewed as “naive.”

The naive Bayes is a probabilistic model. This classifier assumes that each feature in the class is strongly independent (naive). It utilizes features that may be a product of the existence of other features and uses the probability calculation to find out to which class an instance belongs. The naive Bayes theorem is denoted by Formula (1).

(1) $$P\left(Y\left|X\right.\right)=\frac{P\left(X\left|Y\right.\right)P\left(Y\right)}{P\left(X\right)}$$

#### 2.1.3. KNN Retrieval (Phase II)

**Figure 2:**
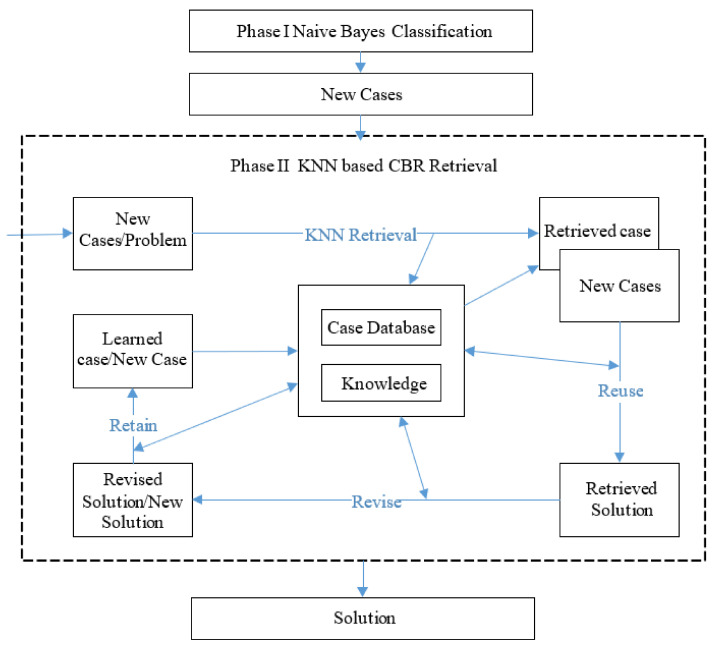
Diagram of KNN retrieval phase.

KNN retrieval is the best approach to select highly similar cases according to their attributes and assigned weight. The second phase of the experiment includes KNN retrieval based on a case-based reasoning system on a Bayesian classification dataset. This phase is the primary phase of the experiment, as shown in Figure 2.

##### Case Retrieval

The KNN retrieval is subdivided into two parts [30]: (1) Selection step: the similarity of patients is computed and sorted, $p\prime \;\in \;P_{L}$, followed by selecting the K similar instances p′. (2) Fusion step: a numeric value is calculated that determines the similarity between the new case and a set of probable classes within the training set $C_{L}$. This step quantifies the probable outcome of a patient’s $\left(p\;\in \;P_{U}\right)$ decision making, $S_{p}$, which is computed as follows in Formula (2):

(2) $$S_{p}=\frac{\sum_{p\prime \in P*K}w_{p\prime }d{(p,\;p\prime )}^{-1}y_{p\prime }}{\sum_{p\prime \in P*K}w_{p\prime }d{\left(p,\;p\prime \right)}^{-1}}$$

where P*K refers to the optimal KNN and the formula contains the labels assigned to the set stored in $P_{L}$, which refers to a considered set in $C_{L}$. $P_{L}$ has the biggest similarity measure with respect to the currently analyzed patient $p\;\in \;P_{U}$. The rest of the variables represent the following: p, new patient; p′, similar patients; and $p\prime \;\in \;P_{L}$, sorting similar patients.

The set of patients’ weights is represented by ${\left\{w_{p^{\prime }}\right\}}_{p\prime \in P_{L}}$, which is designed to validate $\sum_{p\prime \in P_{L}}w_{p^{\prime }}$ and ${\left\{y_{p\prime }\right\}}_{p\prime \in P_{L}}$.

The nearest neighbor retrieval computes the similarity among previously stored cases and new cases based on weight features. The similarity is computed as follows in Formula (3):

(3) $$\mathrm{similarity}\left(\mathrm{CaseI},\mathrm{CaseR}\right)=\frac{\sum_{i=1}^{n}w_{i}\times \mathrm{sim}\left(f_{i}^{I},f_{i}^{R}\right)}{\sum_{i=1}^{n}w_{i}}$$

**Figure 3:**
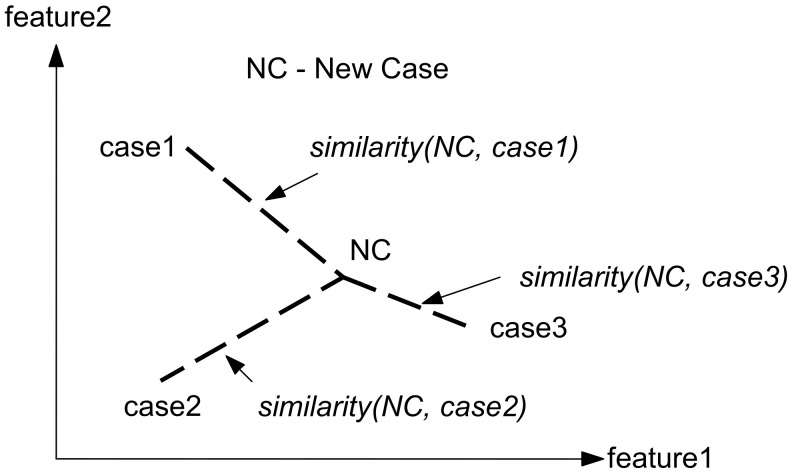
How to find the nearest neighbor of the new case (NC).

This formula shows an effortless computation of nearest neighbor matching, where $w_{i}$ is the importance of the feature weight. Sim represents the similarity function of features, $f_{i}^{I}$ is the value of feature I in the input, and $f_{i}^{R}$ is the value for feature i in the retrieval. An illustration of how nearest neighbor works is shown in Figure 3. In the following two-dimensional space, case3 is chosen as the nearest neighbor since similarity (NC, case3) > similarity (NC, case1) and similarity (NC, case3) > similarity (NC, case2).

#### 2.1.4. The Fusion of ECC (Phase III)

The cases retrieved according to KNN may be more in line with the needs of the machine, especially in terms of retrieval accuracy, but the decision-making behavior of physicians in real-world application scenarios often involves many practical factors which are based on the KNN case retrieval process. The results of machine-learning-based retrieval may not be suitable for the final clinical diagnosis. Therefore, it is necessary to introduce the prior knowledge of ECC in order to obtain a sequence of retrieved cases that is more in line with the logic of the clinical auxiliary diagnosis of breast cancer.

Experts experienced in clinical diagnosis usually consider more practical factors affecting breast cancer diagnosis, such as the content, source, and physician assessment of other similar cases. The aggregation of ECC information can not only compensate for the shortcomings of machine learning models, but also provide some diagnostic assistance to physicians. This will bring informative benefits to our findings and help us discover cases with searches that physicians will trust and that are more in line with their diagnostic habits.

The ECC mentioned in this study include the authority of the source of the case and the evaluation of the case by the doctor. In this paper, the authority of the source of the cases refers to the fact that historical cases come from doctors with different specialties, and these doctors with different specialties result in differences in quality between cases, which specifically include physician’s professionalism and willingness to reveal their real name. The doctor’s evaluation of the cases refers to the scoring and evaluation made by a doctor after using a historical case, and represents the quality of the case.

In order to integrate the ECC features, weights were assigned to the physician’s professionalism, willingness to reveal their real name and case quality score. The method used to determine the weights in this paper is the expert scoring method, which objectively combines the experience of most experts with subjective judgement to provide a reasonable estimation of factors that are difficult to analyse quantitatively. However, each expert has a different background and may score differently, so it is necessary to consider the consistency of the scores graded by experts to compare the consistency of the results obtained by different methods. There are many methods to test for consistency, such as the Kappa test, the ICC intra-group correlation coefficient, and the Kendall’s W coordination coefficient. In this paper, the Kendall’s W coefficient of concordance is used because it is suitable for comparing the consistency of data from multiple groups of doctors on a particular indicator. Therefore, this paper uses the Kendall’s W coefficient of concordance in SPSS software to test the consistency of expert scores for later integration of ECC.

The ECC are integrated into the results of the first stage through the binary harmonic mean method. In contrast to the experimental results that consider the fusion of ECC and the experimental results that do not consider the standard fusion of ECC, we analyze the influence of the ECC on the retrieval results. We then discuss the use of this influence in medical decision making, meaning in management of disease.

The binary harmonic mean method used in this phase refers to the arithmetic average of the reciprocal of n numbers, which has a special penalty mechanism to obtain reasonable averages closer to the smaller values (i.e., valuing the smaller values) among the evaluation scores of different dimensions. In this paper, we want to pay more attention to the characteristics of the case content itself and properly integrate the characteristics of the case sources, so we use the binary harmonic mean method to enable the results to converge more closely to the results we want and thus make the evaluation more accurate. The Formulas (4) and (5) for the specific fusion are shown below.

(4) $$P_{t}{=w}_{i}*x_{i}$$

(5) $$S_{P_{t}}=\frac{2*S_{t}*P_{t}}{S_{t}+P_{t}}$$

where $w_{i}$ represents the weight value of the i th feature in the ECC, $x_{i}$ represents the value of the i th feature in the ECC,$\;P_{t}$ represents the weighted sum of all features of the ECC of the t th case, $S_{t}$ represents the similarity value of the t th case, and $S_{P_{t}}$ represents the final fusion value of the similarity value of the t th case itself and the weighted sum of the ECC features of the t th case.

**Figure 4:**
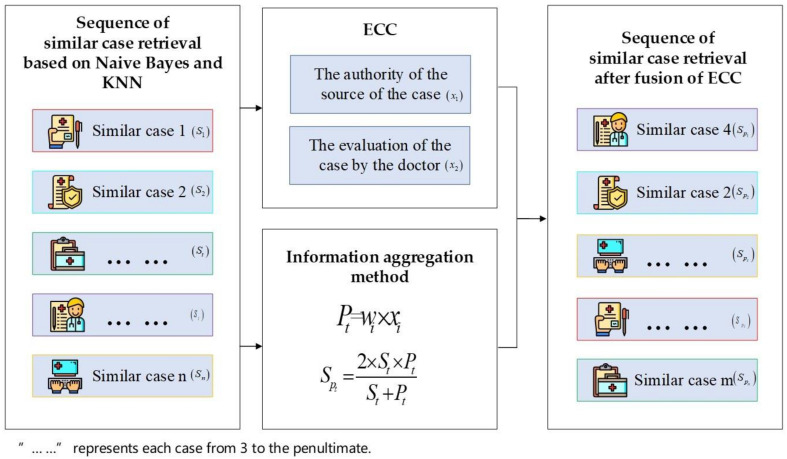
The case solution integration process considering ECC.

Figure 4 shows the ECC integration process.

#### 2.1.5. Case Adoption

Case adoption consists of merely editing the obtained case solution to address a problem of a new case. Adaption is implemented by exclusion, inclusion, replacement, or even alteration of an output (i.e., case solution). After adoption is completed, new cases are updated to the case database, thus providing a possible potential solution option for a new case problem. The assumptions adopted in the phase I experiments contributed to the similarity improvement. Some of the assumptions are linked to breast cancer’s common risk factors, such as the age feature in our datasets.

## 3. Results

In this study, we propose a CBR-ECC model under a new case retrieval framework. This model adopts an algorithm model combining naive Bayes and KNN to search for similar cases, and integrates ECC to gain information. Real datasets are then used to evaluate the performance of this model. Finally, this model is integrated into the PMDSS-BCAD system [31]. We performed a full comparative study of each phase. We compared J48, KNN, and naive Bayes in the first phase, in the second phase we compared KNN with different K values based on a naive Bayes classification, and in the third phase we designed the whole method as a system and compared it with the CDSS system commonly used in hospitals, so our results for both the comparison of each phase and the whole system all have good accuracy and stability.

### 3.1. Phase I

The first phase of the experiments includes classifying the dataset for purposes of labeling. The dataset was divided into a training set and a testing set. The pre-selected classification methods were then implemented. The training data were used to assemble the model, and the testing data were used to examine the performance. A third of the dataset was used for model testing, and the rest is used for training the model. The purpose of applying a classifier is to group two different categories. Accordingly, class one represents patients with breast cancer (malignant breast tumor) and class zero represents patients without cancer (benign breast tumor). The evaluation includes precision, recall, F-measure, and accuracy.

We set four standard metrics: true positive (TP), true negative (TN), false positive (FP), and false negative (FN). TP represents the number of malicious records. TN represents the number of benign cancers, correctly classified. FP represents the number of benign tumors incorrectly diagnosed as malignant tumors. FN represents the number of benign tumors incorrectly classified as malignant tumors.

To build the descriptive model and obtain cases for knowledge representation, three classification models including a J48 decision tree, KNN, and naive Bayes were constructed and evaluated. The classifier with the best overall performance was naive Bayes, as shown in Table 3. From these results, it is clear that naive Bayes has the best overall performance in comparison to the other classifiers, with its accuracy of 95.87%, precision of 94.45%, recall rate of 95.82%, and F-measure of 95.79%.

**Figure 5:**
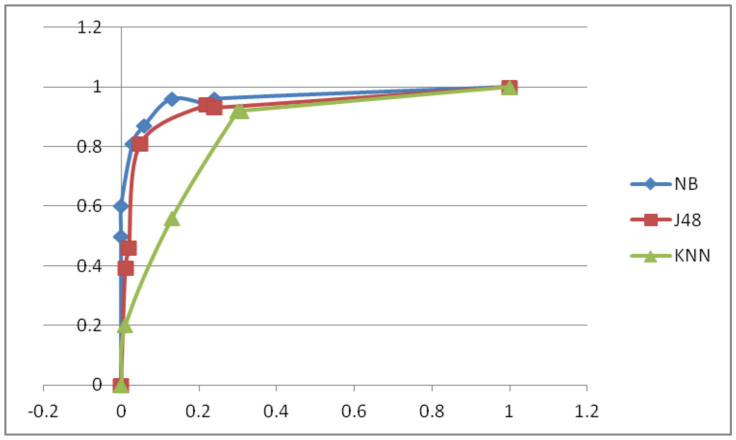
ROC curves of the classfiers used.

The results were also evaluated using the receiver operating characteristic (ROC) curve. This is a visual tool for effectively comparing two or more binary classification models. It compares the sensitivity and false-positive rate of the models. Each point represents a sensitivity/specificity pair that matches a different decision threshold. The area under the ROC curve is the index and basis for comparing model accuracy. It shows how well or badly a parameter distinguishes between two different groups [32]. Generally speaking, a model with a large area is a model with higher accuracy, that is, the model to be selected. In addition, a diagonal orientation is also a good reference method. The closer the ROC curve is to the diagonal, the lower the model accuracy will be. On the contrary, the closer the model is to the upper-left corner of the plot, the more accurate the model is. The naive Bayes classifier proved to be the one with the lowest error rates, as we can clearly see in the ROC curves illustrated in Figure 5. The performance of the J48 decision tree classifier is near the naive Bayes curve. Both of these classifiers clearly outshined the KNN classifier.

### 3.2. Phase II

We then moved to the next phase, which is crucial to the study: the retrieval phase using KNN. The retrieval phase is the most important phase of the PMDSS-BCAD system, and KNN was implemented in the previously classified dataset for purposes of improving the retrieval results. There are two distinct categories in the datasets: class one, representing cases (patients) with a malignant tumor, and class zero, representing patients with a benign tumor. KNN will aid in retrieval by indicating which category the tumor belongs to, whether it is malignant or benign. The KNN algorithm will enable the most optimal selection of similar cases by selecting only cases with the highest similarity rates.

#### 3.2.1. Evaluation of the Performance of KNN

The selection of the optimal value of K for the evaluation performance of KNN completely depends on the data. The chosen value of K directly influences the classifier performance. A large value of K makes the classification boundaries less obvious, decreasing the classifier performance. If the value of K is very small, it may cause over-fitting due to noise within the training set. To single out the most optimal value of K, we performed a cross-fold validation test on the dataset [33].

The right model complexity is the one that generates the minimum generalization error. The estimation of the error is helpful for the algorithm to select the model and find the model with the correct complexity, rather than over-fitting. After assembling the model, it is then implemented to the test set for the prediction of the class label of never-seen records. Generally, it is practical to gauge the execution of a model on its test set since it gives a fair measurement of its speculative error. The precision or error rate registered from the test set can likewise be utilized to analyze the relative execution of various classifiers on a similar domain. Nevertheless, for this to be done, the class names of the test records must be distinguished.

There are four common approaches used to evaluate the performance of a classifier: the holdout method, random sampling, bootstrap, and cross-validation. In our study, cross-validation is used. Data are partitioned into two sets. Parts of the subsets are changed. The training set turns into the test set, and the test set turns into the training set. We call this a twofold cross-validation. The total error is obtained by adding the errors of the two runs. Here, every record is utilized precisely once for training, as well as one time for testing.

The K-fold cross-validation technique sums up this approach by sectioning the information into K parallel estimated partitions. Throughout each run, one of the segments is selected for testing, while whatever is left in the other segments is utilized for training. The method is reused K times, so each parcel is utilized for testing precisely once. The aggregate error is found by adding up the errors for all K runs.

**Figure 6:**
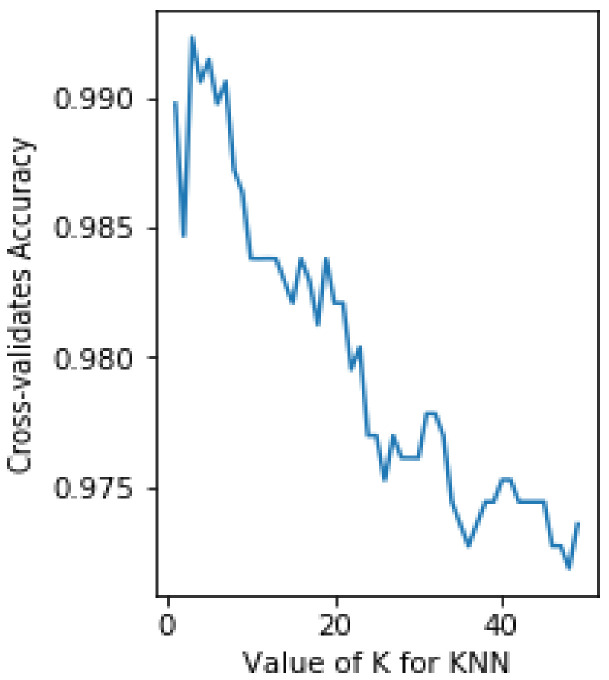
Optimal value of K.

We conducted cross-validation tests to determine the optimal K value of our model. The results show that, as described in Figure 6, the optimal value of K is 2, with a cross-validation accuracy of 99%.

The KNN results are also evaluated in terms of accuracy, precision, recall, and F-measure, as described in Table 4. The results of this analysis prove the efficiency of the KNN classifier when choosing the optimal value of K = 2.

#### 3.2.2. KNN Retrieval Results

KNN is a commonly used CBR retrieval method. It improves the CBR retrieval phase, aiding experts in finding the most similar cases. Below are the cases that will be input into KNN for retrieval after naive Bayes classification. As shown in Table 5, the new case is the case with index 1.

Table 6 shows the similar cases from most similar to least similar, according to the value of similarity in the first row. The results show that the two most similar cases to the new case have the index numbers 1135 and 433, according to the similarity values displayed in the top row.

The KNN retrieval implemented on a previously classified dataset with naive Bayes shows favorable results, and its ROC curve is clearly the one closest to the upper-left corner. By simply implementing a Bayesian classification prior to proceeding with the traditional method, we can obtain good results. However, combining naive Bayes with KNN yields even better results.

This method delivers significantly better results due to the fact that naive Bayes allows a better-structured set capable of easily and efficiently dividing the distinct classes (in our case, the database), allowing a smoother retrieval phase that is further enhanced by KNN, a commonly used retrieval method that has repeatedly proved to be exceptional, and this is particularly true when paired with naive Bayes.

### 3.3. Phase III

In this paper, 10 doctors specializing in breast cancer were separately searched to score the ECC attributes (1–10), and the weight of the mean score obtained for each feature attribute versus the sum of the mean scores of all attributes was used as the weight of that attribute. To ensure the reasonableness of the scoring, this paper conducted a consistency test on the data by SPSS software. Among them, the Kendall’s W = 0.840 for the consistency test, with *p* < 0.001, indicating that the data had good consistency and could be used for experimental analysis. The corresponding $w_{i}$ values were finally obtained: physician professionalism = 0.4, willingness to reveal real name = 0.1, and case quality score = 0.5. After incorporating ECC, the ranking of some similar cases changed, as shown in Table 7. In previous experience, when the personalized recommendation system recommends similar cases to physicians, the primary similarity is ranked by order of magnitude, and the physician may choose the first case or one of the first few similar cases for the proposed solution to assist in the decision-making process. The significance of the integration of ECC in this paper is that the addition of expert empirical knowledge makes the ranking evaluation index more comprehensive, enabling similar cases with lower similarity rankings to be highlighted, cases that might be more important for the doctor’s decision making.

As shown in Table 7, this study initially demonstrates that the incorporation of ECC has a certain influence on the ranking of similar cases. Compared with the previous ranking based on similarity alone, this paper takes into account not only the influence of objective factors (internal attributes of cases), but also the influence of ECC (physician evaluation and case authority), which makes the ranking index more comprehensive and the recommendation results more reasonable.

According to the survey results of 10 breast cancer physicians, we found that the right-hand system in Table 7 (with ECC) was better than the left-hand system (without ECC) in several aspects of user evaluation of the system. The results are shown in Table 8.

### 3.4. Case Adoption

Case adoption refers to the case solution recommended by the system being adopted by the system users, which is the result of the system users reworking the system output according to the recommended system output. It is the process by which the personalized system interacts with physicians and patients. Therefore, whether a case is adopted or not reflects the quality of the personalized system recommendation to a certain extent. To quantitatively reflect the quality of the personalized system recommendations, this paper has designed several variables to evaluate the quality of the personalized recommendation system output from the perspective of doctors and patients: adoption of knowledge, ease of use, participation of physician, usefulness to improve medical quality, satisfaction of use, and intention of continued use [34].

In this paper, 10 breast cancer doctors were asked to rate the above attributes (1–10), and the mean score of each attribute was used as the percentage of participants who said “yes” relative to the total score of the attribute. To ensure the rationality of the scoring, the highest score and the lowest score were removed, and then the data were tested for consistency by SPSS software. The Kendall’s W = 0.743, with *p* < 0.001, for the consistency test of the system assessment scoring without considering ECC, and the Kendall’s W = 0.735, with *p* < 0.001, for the consistency test of the system assessment scoring considering ECC, indicating that the data were highly consistent and can be used for experimental analysis. The corresponding user evaluation results were obtained, as shown in Table 8.

As shown in Table 8, the indicators of the personalized recommendation system changed for both physicians and patients after incorporating ECC. The percentage of participants responding positively to the indicators increased significantly after the integration of ECC.

This further proves that the integration of ECC influences the adoption of similar cases. In this paper, we not only consider the accuracy of the algorithm output, but also take into account the participants’ evaluation of the system, so that the recommended results are more in line with doctors’ habits and better meet the needs of both doctors and patients.

## 4. Discussion

We developed and validated a combined CBR-ECC model in a new case retrieval framework using a dataset from the Maputo Central Hospital in Mozambique. The combined model was integrated into the PMDSS-BCAD system and the capabilities of the system were tested in a real hospital scenario. We conducted this study and evaluated the machine learning algorithm using ROC curves and clinical diagnostic efficiency as criteria. Our preliminary results show that the combined CBR-ECC model has superior diagnostic performance and good confidence. This is reflected in the better performance of our naive Bayes algorithm compared to KNN and J48 decision tree classifiers with an accuracy of 95.87%. Based on the positive and negative case dataset classified by naive Bayes in the initial stage, we selected the KNN model with the best K value to retrieve similar cases and the results showed an accuracy of 99.40%. In addition, we found that the similarity between retrieved cases in the proposed new CBR framework was high after the implementation of KNN, with the minimum distance decreasing all the way down to 0.13. When we fused the diagnostic results obtained by naive Bayes and KNN with ECC, we found that the ranking of similar cases changed, providing preliminary evidence that the addition of ECC had an impact on the ranking of similar cases. Our findings have higher predictive accuracy and higher trustworthiness. Furthermore, the prediction and trustworthiness of our system was improved as new cases were added to the case base. Thus, the proposed case-based breast cancer diagnosis system, which integrates internal and external characteristics of cases, ECC, can help patients through early disease screening, help clinicians select highly accurate and credible treatment strategies, reduce cancer risk in women with extensive demographic information, and improve the quality of medical interventions, making the proposed system a potential tool for breast cancer management in clinical settings.

Breast cancer management requires the combined efforts of medical and nursing professionals as well as technical staff. However, traditional diagnostic methods rely solely on the clinical experience of physicians and are highly susceptible to misdiagnosis. With the continuous development of machine learning in medical research, the diagnostic tools for breast cancer have made a great leap forward. Al et al. [35] reported an accuracy of 93.75% and 88.75% for SVM and KNN on breast cancer diagnostic classification tasks, respectively. Hoque et al. [36] proposed an improved KNN-DK algorithm that was able to achieve 94.9% classification accuracy on a breast cancer database. The design of classifications using KNN, a KNN-improved algorithm, or SVM directly, although it has performed well in terms of performance, may not be logically more appropriate than two more refined classifications, because as far as the seriousness of medical treatment is concerned, we are not committed to a significant improvement in accuracy; we care more about a further reduction in error rate, which represents a reduction. This idea corroborates our first innovation point, which is to use a combined CBR-ECC model, which first uses naive Bayes to separate positive and negative cases and then uses KNN to retrieve more similar cases. Our final detection combination model achieves an accuracy of 99.40%, which is a significant improvement in performance compared to methods in the latest literature.

In addition, traditional breast cancer diagnosis methods only consider the characteristics of the cases themselves, and neglect the impact of external characteristics of the cases on the recommendation results from the perspective of physicians’ trustworthiness within the system. Based on the diagnostic logic of similar reasoning based on previous medical records, we believe that doctors will consider not only the characteristics of breast cancer itself, but also the credibility and assertions from higher authority experts, which will also play a role in the diagnosis process. Therefore, another innovation of this study is that we propose an innovative ECC feature and verify the enhanced effect of the fusion of ECC features on physicians’ adoption of the results.

## 5. Conclusions

In this paper, we developed a combined model of naive Bayes and KNN algorithms taking ECC into account (the CBR-ECC model) and a PMDSS-BCAD system under the framework of new case retrieval. The model and system combine the internal and external characteristics of the case and can provide results with greater similarity rates and a more physician-appropriate outcome, making the adoption process easier for health practitioners when diagnosing breast cancer cases.

Our research is of great significance in both theory and practice. In theory, we implemented naive Bayes, the KNN classifiers, and the ECC fusion method under the new case retrieval framework, which improved the retrieval efficiency of the intelligent diagnosis system for breast cancer. This framework can contribute to setting up an alternative breast cancer diagnosis system based on improvement of KNN case retrieval. In practice, this study will help assess the cognition, attitudes, and practices of the local communities in understanding breast cancer.

However, the proposed approach also has limitations. For example, since having ECC features is an innovative aspect, as presented in our article, we have not found any publicly available dataset with similar properties at this time. There is, therefore, currently no opportunity to test our method on other databases. For future research we will try to collaborate with some hospitals to further focus on the organization of cross-hospital case knowledge and human–machine collaboration. 

## Figures and Tables

**Table 1:** Preview of the final dataset used.

No.	Age	Consistency	Clump Thickness	FNA	Swabs	Class	Specialty of Doctors	Willingness to Reveal Real Name	Case Quality Scoring
1	59	5	1	1	6	1	5	1	8
2	29	5	6	1	5	1	7	1	7
3	53	1	1	1	9	1	6	1	5
……	…..	…..	……	…..	…..	…..	…..	…..	…..
90	33	5	3.5	1	3	1	5	1	10
91	22	2	3	2	5	0	5	1	7
92	18	5	2.8	1	3	0	10	1	8

**Table 2:** Attribute descriptions.

Attribute	Description
Age	The age of the patient; the older they are, the higher the chance of having cancer. The patients are aged from 10 to 95.
Consistency	The consistency of the tumor, which we grouped in order from soft to hard; we used numbers to represent each value, namely: (1) soft; (2) elastic; (3) fibro-elastic; (4) fibrous; and (5) hard.
Clump thickness	The size of the clump (tumor) found in the breast. It varies from 0 to 22 cm.
FNA	The type of material (substance) extracted from fine-needle aspirate (FNA) tissue specimens of breast cancer. It varies from 1 to 5, according to the different variations of FNA.
Swabs	The number of swabs made from the extracted material and displayed on glass blades for analysis. It varies from 1 to 10.
Class	A description of the type of tumor, with 1 for malignant and 0 for benign.
Specialty of doctors	The specialty of the doctors: Factors such as clinical level, patient ratings, and title can be considered and scored from 1 to 10. This is used to distinguish the degree of expertise of the physician from whom the case originated.
Willingness to reveal real name	The willingness of the physician to reveal his or her real name to indicate the credibility of the case source. Willing to reveal is 1, and unwilling is 0.
Case quality scoring	Physician rating of the quality of the recommended cases. It is used to distinguish which case is a better one to study in similar situations. Scores range from 1 to 10.

**Table 3:** Comparative performance analysis of the classifiers.

Evaluation	J48	KNN	Naive Bayes
Accuracy	93.02%	93.19%	95.87%
Precision	95.43%	91.94%	94.45%
Recall	93.23%	93.78%	95.82%
F-measure	94.22%	92.27%	95.79%

**Table 4:** Analysis of the best value of K.

Evaluation	Percentage
Accuracy	99.40%
Precision	98.65%
Recall	98.89%
F-measure	98.90%

**Table 5:** New case.

Index	Gender	Age	Consistency	Clump Thickness	FNA	Set	Swabs	Cells	Class
1	0	29	5	6	1	1	5	1	1

**Table 6:** Most similar retrieved cases.

Index	1135	433	393	683	87	85	616	149	610	1070
Similarity	0.86942889	0.82831559	0.79152888	0.77630098	0.76520699	0.75567393	0.73879154	0.72252997	0.71857019	0.71366795
Gender	0	0	0	0	0	0	0	0	0	0
Age	19	18	45	23	36	20	18	18	37	43
Consistency	3	3	5	2	5	1	2	2	5	2
Clump Thickness	1	3	22	1	3.5	3	1	4	1	5
FNA	1	1	1	1	1	1	1	1	1	1
Set	0	0	1	0	1	1	0	0	1	0
Swabs	3	2	3	5	3	9	3	5	2	2
Cells	4	4	1	4	1	9	9	4	9	1
Class	0	0	1	0	1	0	0	0	0	1

**Table 7:** Recommended sorting of similar cases.

Sorting of Recommended Similar Cases When Not Considering ECC		Sorting of Recommended Similar Cases When Considering ECC	
Index	Sorting Value	Index	Sorting Value
1135	0.86942889	683	0.85843290
433	0.82831559	433	0.82911822
393	0.79152888	87	0.79628764
683	0.77630098	1135	0.78769023
87	0.76520699	393	0.73728556

**Table 8:** The user evaluation results.

Item of Evaluation, Not Considering ECC	Percentage of Participants Answering “Yes”	Item of Evaluation, Considering ECC	Percentage of Participants Answering “Yes”
Adoption of knowledge	75.56%	Adoption of knowledge	91.11%
Ease of use	47.78%	Ease of use	75.00%
Participation of physician	26.67%	Participation of physician	86.67%
Usefulness to improve medical quality	72.22%	Usefulness to improve medical quality	96.11%
Satisfaction of use	61.11%	Satisfaction of use	80.00%
Intention of continued use	48.89%	Intention of continued use	90.56%

## Data Availability

The data presented in this study are available on request from the corresponding author. The data are not publicly available due to the request of respondents.
